# Assessment of Knowledge and Attitude of Asthmatic Patients and Their Caregivers Regarding the Disease Using an Asthma Knowledge Questionnaire in a Tertiary Care Hospital in Chennai

**DOI:** 10.7759/cureus.69832

**Published:** 2024-09-21

**Authors:** Ankita Francis, Elen Abraham, Ghanshyam Verma, Leny Mathew, Sourya Acharya, Sunil Kumar, Nikhil Pantbalekundri

**Affiliations:** 1 Department of Respiratory Medicine, Sree Balaji Medical College and Hospital, Chennai, IND; 2 Department of Neurology, Mar Baselios Medical Mission Hospital, Ernakulam, IND; 3 Department of Medicine, Jawaharlal Nehru Medical College, Datta Meghe Institute of Health Education and Research, Wardha, IND

**Keywords:** assessment, asthma, attitude, disease awareness, inhaler adherence, knowledge, questionnaire

## Abstract

Introduction

Asthma, a significant non-communicable disease impacting all age groups, leads to productivity loss, particularly affecting children and causing disruption within families. This study aims to evaluate the knowledge and attitudes of asthmatic patients and caregivers through questionnaires, focusing on available treatment options.

Aims and objectives

The study aimed to assess the knowledge and attitudes of asthmatic patients and caregivers at a tertiary care hospital in Chennai, Tamil Nadu, India. Objectives included evaluating asthma knowledge using a questionnaire and assessing attitudes towards the disease and available treatment options.

Materials and methods

This cross-sectional study, conducted at the Department of Respiratory Medicine, Sree Balaji Medical College & Hospital, Chennai, focused on bronchial asthma patients and their caregivers in the Outpatient Department (OPD) from May 2021 to November 2022. The study included 150 participants selected randomly based on sample size calculations. Data, collected through a proforma, included demographic variables. Participants' knowledge and attitudes toward asthma were assessed using the Asthma Knowledge and Asthma Attitude questionnaires. Exclusion criteria comprised patients with a history of tuberculosis, chronic obstructive pulmonary disease, interstitial lung disease, recent hemoptysis, or evidence of infective exacerbation.

Results and discussion

Among 150 participants, 44% were females and 56% were males. Asthma knowledge was present in 62.1% of females and 79.8% of males, while 37.9% of females and 20.2% of males lacked understanding. High prevalence rates of breathlessness (89.4% females, 90.5% males), wheezing (92.4% females, 81% males), and cough (77.3% females, 75% males) were noted. The knowledge questionnaire revealed a mean score of 4 out of 8, indicating a moderate understanding with significant gaps. Many lacked knowledge about affected sites, harbored misconceptions about pet contact, and dust exposure, and were uninformed about triggers, symptoms, and treatment. Lack of awareness regarding weather-related exacerbations, smoking effects, and medication purposes was evident. Reluctance towards long-term inhaler use underscored the necessity for comprehensive asthma education among patients and caregivers. Attitude questionnaire scores varied: 17 scored 16, 37 scored 18, 24 scored 19, 35 scored 21, 20 scored 24, and 17 scored 26, averaging 20.6, reflecting generally positive attitudes. The attitude questionnaire, with six questions, highlighted attitudes toward asthma. However, stigma around asthma affecting normal lives (77.3%), fear of lifelong medication use (84%), and embarrassment over public inhaler use (74%) highlighted areas for attitude improvement to enhance therapy compliance and patient outcomes.

Conclusion

Our study highlights concerns among asthma patients and caregivers, particularly those with limited education, regarding treatment side effects. Issues include apprehensions about inhaler therapy's addictive potential, insufficient awareness of asthma triggers, improper inhaler techniques, reluctance towards long-term inhaler use, and embarrassment over public use of inhalers contributing to the study's complexities.

## Introduction

Asthma is a significant non-communicable disease that affects both children and adults. It is the most common chronic disease among children [1]. Asthma is estimated to affect 300 million individuals globally, with increasing prevalence in developing countries associated with increasing cost of treatment and rising burden for patients and the community [2]. Asthma causes a loss in productivity, seriously affects children, and disrupts families, which, in turn, contributes to an unacceptable burden on the healthcare system and results in higher mortality, especially among children [3]. Incidence of asthma in India is around 17.23 million, with an overall prevalence of 2.05% [4]. The total burden of asthma in India is 34.3 million, accounting for 13.09% of the global burden. Asthma accounts for 27.9% of DALY (Disability-adjusted life years) in the Indian population. India has three times higher mortality and more than two times higher DALY compared to the global proportion of asthma burden [5]. The etiology of asthma symptoms includes airway inflammation, resulting in mucus hypersecretion, alteration of the bronchial wall, and bronchial hyperresponsiveness, which is the propensity of smooth muscle cells to respond to unspecific stimuli such as cold air. The disorder’s acute symptoms, also known as “asthma attacks,” are recurrent, cyclic episodes brought on by chronically hyperactive and inflamed airways that impede airflow [6]. Global efforts focus on improving patient education and self-management behavior. The primary reasons for poor prognosis are inadequate education for patients and poor medication adherence [7].

Factors responsible for poor adherence to asthma are medication-related factors and intentional poor adherence. Medication/regimen factors include trouble using the inhaler (e.g. arthritis), complex regimen, availability of a multitude of alternative inhalers, unintentionally failing to comply, misunderstanding instructions, forgetfulness, absence of a daily routine, and expenses. Intentional poor adherence involves the perception that treatment is not necessary, denial or anger about asthma or its treatment, inappropriate expectations, concerns about side effects, dissatisfaction with health care providers, stigmatization, cultural or religious issues, and cost [8].

Healthcare providers are vital in empowering patients with the necessary skills and knowledge to manage asthma. With adequate knowledge of the disease, the treatment regimen will succeed as the patient is aware of appropriate management and how to avoid triggers. Effectiveness of drug therapy highly depends on compliance, which, in turn, affects attitude towards treatment. Patients’ attitudes, beliefs, and expectancies about their plight, coping capacity, and healthcare system influence the disability response to treatment and prognosis. Knowledge attitudes and beliefs of patients towards bronchial asthma are recognized as significant determinants of health behavior. Hence, educating the population can be considered an essential therapy strategy to improve knowledge and attitude towards the disease in patients and caregivers and can influence proper adherence to inhaler therapy and therefore improve therapeutic outcome [9]. The current study aims to assess the knowledge and attitude of asthmatic patients and their caregivers towards the disease using a questionnaire and the available treatment options. We framed two questionnaires for this study: one to assess the knowledge and the other to assess the attitude.

## Materials and methods

The current study is a cross-sectional study that included the patients and their caregivers attending the OPD with bronchial asthma in the Department of Respiratory Medicine, Sree Balaji Medical College and Hospital, Chennai, Tamil Nadu, India. The study period was between May 2021 and November 2022 and included a study population of 150 asthmatic patients and their caregivers. The study participants were selected based on random sampling, and the sample size was calculated statistically. The data collection was carried out with a proforma for demographic variables, and two questionnaires were used to assess the knowledge and attitude of participants towards Asthma. An easily understandable questionnaire was framed. Questions were all closed and thus present with “yes” or “no” options only. The questions were sketched to gain information about the participants' perception of etiology, triggers, symptoms, and treatment regimen. The questions were designed to elicit a short answer or response in a multiple-choice format. The questions dealt with the nature of the illness, natural history, etiology, treatment, and prognosis. No attempt was made to correct a wrong answer or response until the completion of the interview. Two questionnaires were used in the study, namely, the asthma knowledge questionnaire (Table 1) and the asthma attitude questionnaire (Table 2). Each question had a “no” answer and “yes,” where “yes” carried 1 mark and “no” carried 0 mark, with a total score of 8 marks. The attitude of asthma was assessed to determine whether it affects the social life and attitude towards medications used in asthma. Responses were recorded on a 5-point scale, with the highest being strongly agreed (scored as 5), with the total being 30. Higher scores meant a better attitude towards the disease.

**Table 1:** Asthma knowledge questionnare

S. No	Asthma Knowledge Questionnaire	Yes	No
1.	Lungs and air pipes affected		
2.	No disadvantages for asthmatic patients to be in close contact with pets or dust exposure		
3.	Patients have increased asthma attacks in cold climate		
4.	Cough and breathlessness are the most common symptoms in asthma		
5.	Smoking worsens asthma		
6.	Inhalers used for asthma constrict airways		
7.	Medicines used help in reducing inflammation of airways		
8.	Inhaler therapy should be continued even if no symptoms present		

**Table 2:** Asthma attitude questionnaire

S. No	Asthma Attitude Questionnaire	Strongly Agree (5)	Agree (4)	Disagree (3)	Strongly Disagree (2)	Don't know (1)
1.	Asthma does not affect pleasures in life					
2.	Social stigma about asthma is still present					
3.	I feel that all medications prescribed are essential for asthma treatment					
4.	I am confident that regular medications for asthma can help me live a normal life					
5.	I feel that taking inhalers in public is not embarrassing					
6.	I fear that medications for asthma have to be taken lifelong					

Devices used to monitor inhaler technique include dry powder inhaler (DPI), metered dose inhaler (MDI), and breath-actuated inhaler (BAI). For the dry powder device, a Rotacap capsule was inserted into the device. Later, the patients were asked to exhale completely, following which they were asked to grip the mouth piece, seal the lips around it, and take a deep breath. While inhaling, the patients were asked to tilt their heads slightly backwards and were asked to inhale maximum through the mouth. Once the inhalation was complete, they were asked to remove the device from the mouth, hold the breath for 10 seconds or as long as the patient was comfortable, and then breathe out. Following this, the patients were asked to rinse their mouth thoroughly to prevent fungal infections due to oropharyngeal deposition of the medicine. For MDI, the patients were initially asked to shake the device for 5 seconds. Then similar to the inhaler technique, the patients were asked to exhale, following which they were asked to grip the mouth piece, seal the lips around it and take a deep breath. While inhaling, the patients were asked to tilt their heads slightly backwards and inhale maximum through the mouth. The remaining instructions were similar to the previous technique. For BAIs, the prerequisites were similar to the previous technique; however, while the medicine comes out of the inhaler, a click sound is heard, following which the patients were asked to hold their breath for 10 seconds and then relax.

The study included all cases of bronchial asthma and caregivers, aged up to 60 years of age and on inhaler device. The exclusion criteria included patients having ongoing or history of tuberculosis, chronic obstructive pulmonary disease, and interstitial lung disease, patients with recent history of hemoptysis, and patients with evidence of infective exacerbation, e.g., fever, purulent expectoration, loss of smell, loss of taste. Patient information confidentiality was maintained. The study was conducted for one and a half years under the guide's supervision. The methodology of the study was as follows. Patients presenting with asthma and following the inclusion criteria were considered. Patient demographic details were considered, and all contributed history was collected through a proforma. An asthma knowledge questionnaire and an asthma attitude questionnaire were distributed, and the patients were asked to fill them out. The proposal for the study was submitted to the Institutional Human Ethical Committee (IHEC) and approval was obtained (Ref No:002/SBMC/IHEC/2021/1593) from Sree Balaji Medical College and Hospital. All subjects participating in this study adhered to the Ethical Committee guidelines. The results were tabulated and sent to a statistician for analysis. The quantitative data obtained were described using mean and standard deviation. All continuous variables were tested for normality using the Kolmogorov-Smirnov test, and the data were found to be normally distributed. Hence, within and between comparisons were made using parametric tests of significance.

## Results

The present cross-sectional questionnaire study was conducted among 150 study participants, and two questionnaires were used to assess the knowledge and attitude of participants towards asthma. The quantitative data obtained were described using mean and standard deviation. All continuous variables were tested for normality using the Kolmogorov-Smirnov test, and the data were found to be normally distributed. Hence, within and between comparisons were made using parametric tests of significance. The characteristics of the study participants in the current study is given in Table 3. Out of the total 150 study participants, 66 were females and 84 were males, which account for 44% and 56%, respectively.

**Table 3:** Characteristics of the participants in the study

Baseline Characteristics		Frequency (N= 150)	Percentage (%)
Gender			
Females		66	44
Males		84	56
Age range			
18-30 years		46	30.6%
31-50 years		70	46.7%
>51 years		34	22.7%
Education			
Female	10th	35	53
	Graduate	31	47
	Total	66	100
Male	10th	54	64.3
	Graduate	30	35.7
	Total	84	100
Duration of asthma			
Female	<1 year	17	25.8
	1-2 years	11	16.7
	3-5 years	15	22.7
	6-10 years	21	31.8
	10 years	1	1.5
	13 years	1	1.5
	Total	66	100
Male	<1 year	14	16.7
	1-2 years	7	8.3
	3-5 years	25	29.8
	6-10 years	27	32.1
	10 years	5	6
	12 years	1	1.2
	13 years	3	3.6
	14 years	1	1.2
	16 years	1	1.2
	Total	84	100
Knowledge of asthma			
Female	Absent	25	37.9
	Present	41	62.1
	Total	66	100
Male	Absent	17	20.2
	Present	67	79.8
	Total	84	100
Seasonal variation			
Female	Monsoon	30	45.5
	Summer	4	6.1
	Winter	32	48.5
	Total	66	100
Male	Monsoon	19	23.8
	Summer	19	23.8
	Winter	46	52.4
	Total	84	100
Symptomatology			
Breathlessness			
Female	Absent	7	10.6
	Present	59	89.4
Male	Absent	8	9.5
	Present	76	90.5
Wheeze			
Female	Absent	5	7.6
	Present	61	92.4
Male	Absent	16	19
	Present	68	81
Cough			
Female	Absent	15	22.7
	Present	51	77.3
Male	Absent	21	25
	Present	63	75
Family history of asthma			
Female	Absent	7	10.6
	Present	59	89.4
	Total	66	100
Male	Absent	12	14.3
	Present	72	85.7
	Total	84	100
Physical activity limitation			
Female	Absent	5	7.6
	Present	61	92.4
	Total	66	100
Male	Absent	6	7.1
	Present	78	92.9
	Total	84	100
History of allergy			
Female	Absent	10	15.2
	Present	56	84.8
	Total	66	100
Male	Absent	12	14.3
	Present	72	85.7
	Total	84	100

While questioned about the history of breathlessness among study participants, a total of 89.4% (n=59) of female participants and 90.5% (n=76) of male participants had a history of breathlessness; however, 10.6% (n=7) of female participants and 9.5% (n=8) of male participants had no history of breathlessness. While questioned about the knowledge of asthma among study participants, a total of 62.1% (n=41) of female participants and 79.8% (n=67) of male participants knew about asthma; however, 37.9% (n=25) of female participants and 20.2% (n=17) of male participants did not know about asthma or its treatment plan.

While questioned about the history of seasonal variation among study participants, a total of 45.5% (n=30) of female participants and 23.8% (n=19) of male participants had history of seasonal variation in monsoon, 6.1% (n=4) of female participants and 23.8% (n=19) of male participants had history of seasonal variation in summer, 48.5% (n=32) of female participants and 52.4% (n=46) of male participants had history of seasonal variation in winter.

While questioned about the history of breathlessness among study participants, a total of 89.4% (n=59) of female participants and 90.5% (n=76) of male participants had a history of breathlessness; however, 10.6% (n=7) of female participants and 9.5% (n=8) of male participants had no history of breathlessness. While questioned about the history of wheezing amongst study participants, a total of 92.4% (n=61) of female participants 81% (n=68) of male participants had a history of wheezing; however, 7.6% (n=5) of female participants and 19% (n=16) of male participants had no history of wheeze. While questioned about the history of cough amongst study participants, 77.3% (n=51) of female participants and 75% (n=63) of male participants had a history of cough; however, 22.7% (n=15) of female participants and 25% (n=21) of male participants had no history of cough.

We evaluated the other common variables during history-taking, such as family history of asthma, physical activity limitation, history of allergy, asthma treatment measures undertaken by the study participants, and technique of inhaler use. Moving on to the next parameter, the medications taken by the study participants, they were given options, namely, BAI, DPI, MDI, tablets, and nebulizers. Considering the female participants, 10.6% used BAI, 27.3% used DPI, 12.1% used MDI, 39.4% used nebulizers, and, similar to BAI, only 10.6% used tablets as medication for asthma. Considering the male participants, 9.5% used BAI, 20.2% used DPI, 19% used MDI, and 28.6% used nebulizer therapy; however, 22.6% used tablets as medication for asthma (Table 4).

**Table 4:** Asthma treatment measures among study participants.

Asthma treatment measures	Female	Male
Breath-actuated inhalers	10.6% (n=7)	9.5 (n=8)
Dry powder inhalers	27.3% (n=18)	20.2% (n=17)
Metered dose inhalers	12.1% (n=8)	19% (n=16)
Nebulizer	39.4% (n=26)	28.6% (n=24)
Tablets	10.6% (n=7)	22.6% (n=19)
Total	100% (n=66)	100% (n=84)

Evaluation of the most necessary parameter, namely, the technique of inhaler use, there was a positive response of 71.2% from female participants and 75% from male participants, with a negative response, where the participants did not know how to use the inhalers, as given by 25.8% of female participants and 16.7% of male participants. However, 3% of female participants and 8.3% of male participants did not have any idea about the inhaler technique (Table 5).

**Table 5:** Technique of inhaler use among study participants.

Technique of inhaler use			
Gender	Response	Frequency (N=150)	Percentage (%)
Females	Positive response	47	71.2
	Negative Response	17	25.8
	No response	2	3
	Total	66	100
Males	Positive response	63	75
	Negative response	14	16.7
	No response	7	8.3
	Total	84	100

For question 1 of the asthma knowledge questionnaire, which states that “Lungs and air pipes affected?” 68 (45.3%) answered “Yes” and 82 (54.6%) answered “No,” and this difference was said to be statistically significant (p<0.001). For question 2, which states that “No disadvantages for asthmatic patients to be in close contact with pets or dust exposure,” 126 (84%) answered “Yes” and 24 (16%) answered “No,” and this difference was said to be statistically significant (p<0.001). For question 3, which states that “Patients have increased asthma attacks in a cold climate”, 71 (47.3%) answered “yes” and 79 (52.6%) answered “no,” and this difference was said to be statistically significant (p<0.001). For question 4, which states that “Cough and breathlessness are the most common symptoms in asthma,” 74 (49.3%) answered “Yes” and 76 (50.6%) answered “No,” and this difference was said to be statistically very highly significant (p<0.001). For question 5, which states that “Smoking worsens asthma,” 39 (26%) answered “Yes” and 111 (74%) answered “No,” and this difference was said to be statistically very highly significant (p<0.001). For question 6, which states that “Inhalers used for Asthma constrict airways,” 9 (6%) answered “Yes” and 141 (94%) answered “No,” and this difference was said to be statistically very highly significant (p<0.001). For question 7, which states that “Medicines used help in reducing inflammation of airways,” 27 (18%) answered “Yes” and 123 (82%) answered “No,” and this difference was said to be statistically very highly significant (p<0.001). For question 8, which states that “Inhaler therapy should be continued even if no symptoms present”, 8 (5.3%) answered “Yes” and 142 (94.6%) answered “No,” and this difference was said to be statistically very highly significant (p<0.001).

Out of 150 participants in the study, 20 were able to answer seven questions with correct answers, 15 were able to answer six questions with correct answers, 54 were able to answer five questions with correct answers, 59 were able to answer four questions with correct answers, and two were able to answer only two questions with correct answers. This thus proves that the mean of the knowledge questionnaire score assessed was 4 out of 8 (Table 6).

**Table 6:** Results of asthma knowledge questionnaire

S. No.	Questions	Yes (1 mark)	No (0 mark)	P-value
1	Lungs and air pipes are affected	68 (45.3%)	82 (54.6%)	<0.001
2	No disadvantages for asthmatic patients to be in close contact with pets or dust exposure	126 (84%)	24 (16%)	<0.001
3	Patients have increased attacks in cold climate	71 (47.3%)	79 (52.6%)	<0.001
4	Cough and breathlessness are the most common symptoms in asthma	74 (49.3%)	76 (50.6%)	<0.001
5	Smoking worsens asthma	39 (26%)	111 (74%)	<0.001
6	Inhalers used for asthma constrict airways	9 (6%)	141 (94%)	<0.001
7	Medicines used help in reducing the inflammation of airways	27 (18%)	123 (82%)	<0.001
8	Inhaler therapy should be continued even if no symptoms present	8 (5.3%)	142 (94.6)	<0.001

Concerning question 1 of the asthma attitude questionnaire, which states that “Asthma does not affect pleasures in life,” 34 (22.66%) answered “Yes” and 116 (77.3%) answered “No,” and this difference was said to be statistically significant (p<0.001). For question 2, which states that “Social stigma about asthma is still present,” 114 (76%) answered “Yes” and 36 (24%) answered “No,” and this difference was said to be statistically significant (p<0.001). For question 3, which states that “I feel all medications prescribed are essential for asthma treatment,” 117 (78%) answered “Yes” and 33 (22%) answered “No,” and this difference was said to be statistically significant (p<0.001). For question 4, which states that “I am confident regular medications for Asthma can help live a normal life,” 87 (58%) answered “Yes” and 63 (42%) answered “No,” and this difference was said to be statistically very highly significant (p<0.001). For question 5, which states that “I feel taking Inhalers in public is not embarrassing,” 39 (26%) answered “Yes” and 111 (74%) answered “No,” and this difference was said to be statistically very highly significant (p<0.001). For question 6, which states that “I fear medications for Asthma have to be taken lifelong,” 127 (84%) answered “Yes” and 23 (15.3%) answered “No,” and this difference was said to be statistically very highly significant (p<0.001).

Out of 150 participants in the study, 17 were able to get a score of 16, 37 were able to get a score of 18, 24 were able to get a score of 19, 35 were able to get a score of 21, 20 were able to get a score of 24, and 17 were able to get a score of 26. This thus proves that, on average, the attitude questionnaire score assessed was 20.6 (Table 7).

**Table 7:** Results of asthma attitude questionnaire

S. No	Questions	Yes (Agree=4)	No (Disagree=2)	P-value
1.	Asthma does not affect pleasures in life	34 (22.66%)	116 (77.33%)	<0.001
2.	Social stigma about asthma is still present	114 (76%)	36 (24%)	<0.001
3.	I feel that all medications prescribed are essential for asthma treatment	117 (78%)	33 (22%)	<0.001
4.	I am confident that regular medications for asthma can help live a normal life	87 (58%)	63 (42%)	<0.001
5.	I feel that taking inhalers in public is not embarrassing	39 (26%)	111 (74%)	<0.001
6.	I fear that medications for asthma have to be taken lifelong	127 (84.6%)	23 (15.3%)	<0.001

## Discussion

In general, the appropriate management of asthma depends on health education. Childhood asthma exacerbations can be avoided by learning more about the condition, recognizing the symptoms and triggering circumstances, and taking the appropriate precautions between attacks [10]. However, knowledge alone will not help control the disease; the prognosis is significantly improved when combined with behavioral therapy. Patients who dislike taking medication will be at greater risk for severe episodes of breathlessness [11]. According to previous studies, there should be a better understanding of attitudes towards medications, which will be helpful for good patient adherence to treatment plans. Asthmatic patients are more prone to anxiety and depression due to the poor attitude of family, which, in turn, affects compliance with medication [12].

The current study revealed that parents of asthmatic children had insufficient knowledge regarding asthma management. The most important reason is falsified information or knowledge about the disease process, which leads to multiple problems related to asthma management. Considering the prevalence of asthma in adolescents and children, air pollution and urbanization were quoted as the prime reasons. In this study, asthmatic patients and caregivers need more knowledge about the disease's etiology and management and clarification about the illness and treatment plan. This should be overcome by increasing awareness about the disease and the management protocols. The evidence of treatment success rate depends on early knowledge of the disease, early recognition of the symptoms, and provision of appropriate treatment. Secondly, attitude towards the treatment plan should be considered, and the patient’s consent should be considered [13]. Due to the globally increasing rate of the condition, patients are ignorant about the disease and refuse to accept the diagnosis due to poor knowledge about the disease's etiology and treatment [14].

Patients and caregivers still need to learn about inhaler use and its continuation in patients without symptoms. Inhaler therapy is the first line of treatment in developed countries; however, its acceptance in our country is poor [15]. There are three types of inhaler therapy, namely DPI, which accounted for 53% in our study, MDI, which accounted for 41% in our study, and BAP, which accounted for 6% in our study. BAI was less preferred due to less oropharyngeal deposition, and therefore, the patients had inadequate feeling of inhaler therapy. In case of acute exacerbations of asthma, people preferred nebulization and injectable corticosteroids for quick relief only and did not prefer hospital admission [16]. The proper and gold standard inhaler technique was seen only in 46% of patients. In our study, every patient’s inhaler technique was monitored at our OPD, and the result was tabulated. For the dry powder device, a Rotacap capsule was inserted into the device. Later, the patients were asked to exhale completely, grip the mouthpiece, seal the lips around it, and take a deep breath. While inhaling, the patients were asked to tilt their heads slightly backwards and were asked to inhale maximum through the mouth. Once the inhalation was complete, they were asked to remove the device from the mouth, hold the breath for 10 seconds or as long as they were comfortable, and then breathe out. Following this, the patients were asked to rinse their mouth thoroughly to prevent fungal infections due to oropharyngeal deposition of the medicine. For MDI, the patients were initially asked to shake the device for 5 seconds. Then, similar to the inhaler technique, the patients were asked to exhale, grip the mouthpiece, seal the lips around it, and take a deep breath. While inhaling, the patients were asked to tilt their heads slightly backwards and inhale maximum through the mouth [17]. The remaining instructions were similar to the previous technique. For BAIs, the prerequisites were similar to the previous technique; however, while the medicine comes out of the inhaler, a click sound is heard, following which the patients were asked to hold their breath for 10 seconds and then relax. It was hence suggested that the correct use of the inhaler was the best way to prevent frequent exacerbations and control the disease [18]. A similar study to assess the level of asthma knowledge and attitudes among patients and caregivers in a tertiary care setting indicated that patients with higher levels of education were more knowledgeable about asthma management, whereas caregivers often lacked sufficient knowledge, impairing their ability to effectively assist patients. Additionally, the study observed that attitudes towards managing asthma were shaped by both education and previous encounters with the disease [19]. The effect of specific educational interventions on asthma knowledge and management among patients and caregivers was examined in another research. The results showed that well-organized educational initiatives notably enhanced both the understanding and attitudes regarding asthma management. Individuals and caregivers who took part in these initiatives demonstrated improved compliance with treatment plans and better handling of asthma symptoms. The study's findings emphasized the importance of continuous education and support in enhancing asthma outcomes [20]. The connection between asthma knowledge and asthma control in patients and caregivers was examined in a similar research. The results indicated that greater asthma knowledge was linked to improved asthma control. The study revealed notable deficiencies in understanding the proper technique of inhaler use and the significance of adhering to prescribed treatments [21]. A different study with the same design evaluated the understanding, beliefs, and behaviors regarding asthma among both patients and their caregivers. It revealed that while overall awareness of asthma symptoms and triggers was satisfactory, there were notable deficiencies in comprehending asthma treatment and medication utilization. The study emphasized the necessity for tailored educational initiatives to enhance asthma management practices and bolster appropriate medication use. Attitudes toward asthma management varied, with some caregivers holding pessimistic views that adversely impacted their adherence to treatment regimens [22]. Thus, teaching and educating the patients and caregivers about the correct inhaler technique to control the disease is necessary.

The study's limitations include the fact that it is a cross-sectional study, which only provides a snapshot of the participant’s knowledge and attitudes at a certain point in time, which does not allow the assessment of changes over time or the identification of causal links. The study included 150 randomly selected participants, which may not apply to the larger population of asthma patients in Chennai or India. The study was carried out at a single tertiary care hospital, which may have introduced selection bias because patients who visit such facilities may have more complex conditions or have better access to healthcare information. The questionnaire employed in the study featured closed-ended questions, which may have missed the nuances of the participants’ comprehension or opinions. The design did not allow for correcting incorrect responses during the interview, which could have provided an opportunity for instant education. Because the study did not involve an intervention to enhance knowledge or attitudes, it cannot provide evidence to address the reported gaps in understanding and positive attitudes about asthma management. The study appears to place a strong emphasis on inhaler therapy, which, while necessary, may not reflect the complete range of asthma management tactics and patient attitudes toward other treatment options. While the study acknowledges adhering to ethical principles, it does not detail the study’s possible influence on participants’ emotional well-being or any precautions taken to prevent any negative impacts of discussing asthma and its impact on their lives. These limitations indicate that, while the study provides valuable insights into asthma patients' and caregivers' knowledge and attitudes at a specific hospital, there is still room for future research using different methodologies, more extensive and more diverse samples, and interventions to improve asthma management and education.

## Conclusions

Our findings suggest that patients with asthma and their caregivers, particularly those with lower education levels, had concerns about the adverse effects of treatment, addiction to inhaler therapy clubbed with a lack of awareness about triggers in asthma, correct inhaler techniques, and hesitancy to use inhalers for a long term, which all complicated the outcome. Therefore, educating patients and their caregivers about the disease and shifting their attitude towards the same in a positive direction to avail early treatment and minimize recurrence will reduce the disease burden on the healthcare system, resulting in lower mortality rates.

## References

[1] Network GA (2024). The Global Asthma Report, Auckland, New Zealand. http://globalasthmareport.org/2018/index.html.

[2] Braman SS (2006). The global burden of asthma. Chest.

[3] Jindal SK, Aggarwal AN, Gupta D (2012). Indian study on epidemiology of asthma, respiratory symptoms and chronic bronchitis in adults (INSEARCH). Int J Tuberc Lung Dis.

[4] GBD Compare. Viz Hub . (2021, June 30 (2024). GBD Compare. Viz Hub. https://vizhub.healthdata.org/gbd-compare/.

[5] Chapman SJ, Grace R, Shrimanker R (2021). Asthma. Oxford Handbook of Respiratory Medicine. 4 edn. Oxford Medical Handbooks.

[6] Gamble J, Stevenson M, McClean E, Heaney LG (2009). The prevalence of nonadherence in difficult asthma. Am J Respir Crit Care Med.

[7] Gaude G, Fernandes N, Avuthu S (2015). Assessment of knowledge and attitude of bronchial asthma patients towards their disease. J Evol Med Dent Sci.

[8] Ma J, Sun X, Wang X, Liu B, Shi K (2023). Factors affecting patient adherence to inhalation therapy: an application of SEIPS Model 2.0. Patient Prefer Adherence.

[9] Martin J, Townshend J, Brodlie M (2022). Diagnosis and management of asthma in children. BMJ Paediatr Open.

[10] Kew KM, Nashed M, Dulay V, Yorke J (2016). Cognitive behavioural therapy (CBT) for adults and adolescents with asthma. Cochrane Database Syst Rev.

[11] Alqarni AA, Aldhahir AM, Siraj RA (2024). Asthma medication adherence, control, and psychological symptoms: a cross-sectional study. BMC Pulm Med.

[12] Papi A, Blasi F, Canonica GW, Morandi L, Richeldi L, Rossi A (2020). Treatment strategies for asthma: reshaping the concept of asthma management. Allergy Asthma Clin Immunol.

[13] Singh S, Salvi S, Mangal DK (2022). Prevalence, time trends and treatment practices of asthma in India: the Global Asthma Network study. ERJ Open Res.

[14] Ribó P, Molina J, Calle M (2020). Prevalence of modifiable factors limiting treatment efficacy of poorly controlled asthma patients: EFIMERA observational study. NPJ Prim Care Respir Med.

[15] Alangari AA (2014). Corticosteroids in the treatment of acute asthma. Ann Thorac Med.

[16] Ferrante G, La Grutta S (2018). The burden of pediatric asthma. Front Pediatr.

[17] Dhabalia S, Acharya S (2021). Bronchial asthma in the era of COVID-19. J Pharm Res Int.

[18] G J, Abraham E, Verma G (2023). Association of asthma with patients diagnosed with metabolic syndrome: a cohort study in a tertiary care hospital. Cureus.

[19] Sabaté E, Verbeek P (2012). Asthma knowledge and attitudes among patients and caregivers in a tertiary care hospital. Clin Respir J.

[20] Kumar S, Sharma R (2019). Impact of patient and caregiver education on asthma management: a study in a tertiary care center. Int J Environ Res Public Health.

[21] Bong J, Kim S (2020). Assessment of asthma knowledge and its relation to asthma control: a patient and caregiver perspective. J Asthma.

[22] Bheekie A, Mansoor S (2013). Knowledge, attitudes, and practices regarding asthma among patients and caregivers: a review. J Asthma.

